# Notes on Methods of Preventing Decay

**Published:** 1902-12

**Authors:** J. Morgan Howe


### NOTES ON METHODS OF PREVENTING DECAY.[1]

BY J. MORGAN HOWE, M.D.S., M.D.

The means suggested for preventing decay that has recently received most attention is that which has been designated by the phrase, "Extension for prevention." I understand the practice, as described and taught, to be a procedure, in the preparation of cavities, that removes sound enamel and dentine in enlarging their area, so that gold or filling-material may be substituted for such dental tissues. It does not refer to the cutting away of partly disintegrated or undermined enamel and dentine that more or less surrounds all cavities. This is removed without reference to the question of "prevention" in adjacent tissues that are still sound.

Dr. Black says, in describing the tendency of certain areas of teeth surfaces to decay,[2] "The principle is established of so extend-

[1] Read before The New York Institute of Stomatology, June 3, 1902.

[2] The Technical Procedures in filling Teeth, 1899.

ing the cavity outline as to include the area of the surface which . . . is especially liable to decay in the future. This is called extension for prevention. It applies to all smooth surface cavities." These brief sentences seem to contain the gist of the practice about which so much has been written, and I wish to add only a little to the discussion, to indicate wherein I think such sacrifice of tissue as yet sound is unjustifiable and illogical. If I repeat what others have already said, I can only hope it will bear repetition.

In regard to any cavity, the area of decayed tissue is in each case more vulnerable than that remaining sound; otherwise it would not be decayed; and when all the dentine and enamel that are affected by the disintegrating process have been removed, neighboring enamel may reasonably be expected to continue sound if the conditions remain the same. It is not necessary to assume that such contiguous surfaces will decay, especially as it will probably be within the patient's power, by greater attention to cleanliness, to make the conditions more favorable than they have been.

The advocates of "extension for prevention" propose to bring about cleanliness of enamel by substituting gold for enamel in those locations less accessible to means of friction, and by leaving enamel only in so-called "self-cleansing" or easily brushed areas. But if the claim is granted that the enamel remaining sound adjacent to a cavity has proved itself less liable to decay than that already destroyed, with the conditions remaining the same, the sacrifice of such sound tissue because it may decay certainly seems unjustifiable.

The principal factor in decay is the susceptibility of the individual. Dr. Black, in reference to this fact, says,[1] "At the present time we are unable to control in any direct way this principal factor, susceptibility; in prophylactic treatment our attention must be directed to the removal, modification, or improvement of those conditions, giving opportunity for its manifestations." To this I think we will assent, but treatment which does not affect the principal factor in the process should be far less destructive of the tissue it is desired to save than is the one under consideration. All the sacrifice of sound enamel and dentine is made to put an imperishable material in its place, where there is some difficulty in keeping the surface clean. But many such surfaces do not decay. So far as

[1] Operative Dentistry, Pathology, and Bacteriology, 1901, p. 270.

I know no suggestions have been made for discriminating in the selection of teeth on which to practise "extension." "It applies to all smooth surface cavities."

When applied, then, to teeth decayed on the proximal surface, and also on the labial or buccal faces, there would be little of the tooth remaining. The æsthetic sense of the present time regards the conspicuous showing of gold in teeth as highly objectionable. "Extension for prevention" and porcelain inlays seem to belong to different periods. The practice of "extension" on proximal surfaces, as taught, does not remove the enamel always most liable to decay. In my own observation of incisor and cuspid teeth it has very frequently happened that decay has occurred at the incisive margin of proximal fillings, when the gingival enamel has remained sound, although considerable of it has been exposed between the filling and the gum. This fact has been the subject of repeated observations during the two years last past, while "extension" has been so much discussed.

I am convinced, too, that in the treatment of the bicuspids and molars, and on all surfaces, accurate mapping out of the portion of the tooth most liable to decay in the future cannot be done so as to justify "extension" in the sense of its present advocates. We are indebted, however, to Dr. Black and his followers, for the agitation of the subject directs our attention, among other things, to the need of extending the margins of cavities in proximal surfaces to make the margins free of contact with the adjacent teeth, when proper contour of filling has been made. I have been observing more carefully, as no doubt you all have done, during the years that "extension" has been under discussion, and I am sure I have seen many proximal surfaces decay next to otherwise good fillings, for lack of such extension and contour. On the other hand, I have been convinced by the endurance of enamel between the gingival margin of fillings and the gum line, when decay had taken place elsewhere, that the sacrifice of the sound tissue referred to, as advocated by the apostles of "extension," to carry the margin of the filling under the gum, would have been an unnecessary and damaging proceeding. The experience and observation of one practitioner does not go far, but all who remember the history of practice will be conservative, I am sure, about adopting methods that involve destruction of tissue.

More than thirty years ago Dr. Robert Arthur devised a system of "Treatment and Prevention of Decay of the Teeth," and these words were the title of a book he published to explain and advocate his method. This consisted essentially in cutting the teeth away from each other, shaping them so as to leave points of contact only at the buccal or labial side near the gum.

He assumed that the enamel and dentine near the gum, left in a projecting point, would remain sound. The proximal surfaces of incisors were sliced off so as to make a V, opening lingually. I have seen incisor teeth so treated with success, and without disfigurement. But Dr. Arthur's system was to be applied to all teeth, bicuspids and molars as well as incisors. He convinced many of our best practitioners of the validity of his method, and dreadful was the havoc and mutilation they effected with chisel and disk. The spaces made between the masticating teeth were a source of great discomfort to the victims, decay was not prevented, and dentists who began the practice with enthusiasm abandoned it after a while, as completely as they dropped copper amalgam after the fervor of early acceptance passed off. There must have come to the minds of many, after the experience with Arthur's method, an appreciation of the impropriety and folly of ruthlessly cutting away so large a proportion of sound tissue, whose salvation they really desired. I was fortunately restrained from falling into the fashion of that time to any great extent by the experience of having the "treatment" with a disk—run by the newly introduced dental engine—applied to some of my own molars. The discomforts I endured in masticating afterwards gave me pause. But there is, after all, some merit in the Arthur method when applied to the incisors—and possibly to the cuspids—with skill and discrimination. I think, although I have not resorted to it in many years, it would be less destructive and more promising of good results than "extension" as taught now.

Arthur's object in slicing off so much of the teeth was to facilitate keeping the surfaces clean and polished. But in addition he wrote that thin tape should be passed several times a day briskly over the surfaces "so as absolutely to guard against the retention upon them of any extraneous substances." "No operator," he said, "will hold himself in any way responsible for an unfavorable result in the cases of persons who are so careless as not to give proper attention to the cleanliness of their teeth." He appears not to have thought of the good chance there would have been for normal and unmutilated teeth that received such care as he prescribed. The revival of interest in polishing the surfaces of the teeth is a fortunate pointer, bringing us back to methods in use before Arthur devised his method of facilitating cleanliness by making artificial spaces.

We are hardly justified in hoping for a speedy solution of the problem of how to control the main factor, susceptibility; yet we may expect it will be possible in the future to produce such changes in the oral fluids as to overcome or modify the predisposition to decay. In the mean time all are agreed that cleanliness of the surfaces of the teeth continues to be our main reliance in efforts to prevent its inception. The question is, Shall we seek to bring about cleanliness by such operative procedures as Dr. Arthur advocated, or as Dr. Black and his followers are now teaching?

We can readily understand, I think, since Dr. Williams has shown how decay begins under the adhering *mucous plaque,* why antiseptic lotions have had so little effect in preventing decay, and can see the need of constantly rubbing off such films from enamel surfaces. We have, however, little on which to congratulate ourselves as a specialty if progress in prophylaxis must be marked by resort to methods involving great destruction of tissue. The history of practice, too, within the memory of many of us may teach us caution in adopting, without due consideration and trial, ideas or methods having authority behind them. We readily recall how much ill-considered work was done in the indiscriminate extraction of teeth merely to make room for those remaining, in the capping of pulps with oxychloride of zinc, in the adoption of copper amalgam, and in the purchase and use of electric batteries for cataphoresis. All these things became fads. They had enough reasonableness to support an argument in their favor, but there was too ready an acceptance because of the authority recommending them. A lesson appropriate to the present time seems to be that every practitioner should use the known facts in regard to the beginning of decay to help him to do a good deal of thinking on his own account concerning prevention, and to hold him back from being too easily led by others.

